# Spinocerebellar Ataxia type 29 in a family of Māori descent

**DOI:** 10.1186/s40673-019-0108-3

**Published:** 2019-10-12

**Authors:** Kathie J. Ngo, Gemma Poke, Katherine Neas, Brent L. Fogel

**Affiliations:** 1 Program in Neurogenetics, Department of Neurology, David Geffen School of Medicine, University of California Los Angeles, 695 Charles E. Young Drive South, Gonda Room 6554, Los Angeles, CA 90095 USA; 2 Genetic Health Service NZ, Wellington, New Zealand; 3 Department of Human Genetics, David Geffen School of Medicine, University of California Los Angeles, Los Angeles, CA 90095 USA

**Keywords:** Cerebellar Ataxia, Spinocerebellar Ataxia, SCA29, Neurogenetics, Gait disorders/ataxia, Māori, Genetic testing

## Abstract

**Background:**

Mutations in the Inositol 1,4,5-Trisphosphate Receptor Type 1 (*ITPR1)* gene cause spinocerebellar ataxia type 29 (SCA29), a rare congenital-onset autosomal dominant non-progressive cerebellar ataxia. The Māori, indigenous to New Zealand, are an understudied population for genetic ataxias.

**Case presentation:**

We investigated the genetic origins of spinocerebellar ataxia in a family of Māori descent consisting of two affected sisters and their unaffected parents. Whole exome sequencing identified a pathogenic variant, p.Thr267Met, in *ITPR1* in both sisters, establishing their diagnosis as SCA29.

**Conclusions:**

We report the identification of a family of Māori descent with a mutation causing SCA29, extending the worldwide scope of this disease. Although this mutation has occurred de novo in other populations, suggesting a mutational hotspot, the children in this family inherited it from their unaffected mother who was germline mosaic.

## Introduction

The autosomal dominant spinocerebellar ataxias (SCAs) are a heterogeneous group of neurodegenerative disorders that cause cerebellar ataxia and degeneration of the cerebellum and brainstem. These genetic diseases have nearly 50 known subtypes characterized with extra-cerebellar central nervous system manifestations varying by specific genetic type [1]. Spinocerebellar ataxia type 29 (SCA29) is a rare congenital-onset autosomal dominant non-progressive cerebellar ataxia caused by mutations in the inositol 1,4,5-triphosphate receptor type 1 (*ITPR1*) gene characterized by early-onset hypotonia, gross motor delay, and mild cognitive impairment [2–6]. Distinct mutations in the same gene are also associated with SCA type 15 and Gillespie syndrome [1, 4].

The Māori are the indigenous people of New Zealand, currently representing approximately 15% of the total population of the country (http://worldpopulationreview.com/countries/new-zealand/). To date, there is limited genomic information from this population publically available, and no comprehensive analysis of spinocerebellar ataxia causes has yet been performed. Here, we describe the genetic analysis of two siblings in a family of Māori descent presenting with congenital-onset non-progressive ataxia by whole exome sequencing (WES).

## Case report

**Fig. 1:**
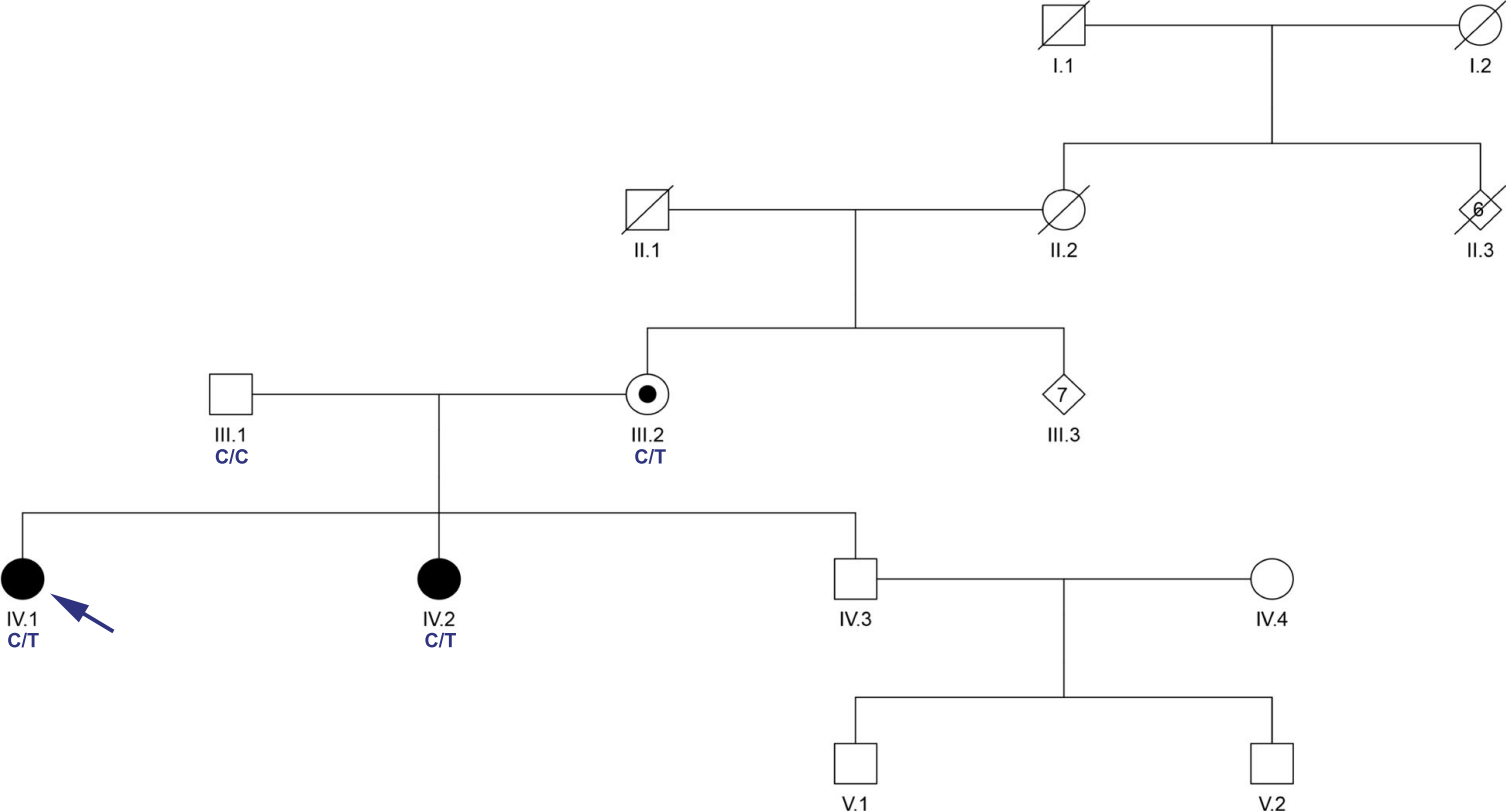
Family Pedigree. Pedigree of the family presented in this report. Proband is indicated by an arrow. Shaded symbols represent affected individuals. A dot indicates germline mosaicism. Genotypes of the c.800 position in *ITPR1* are shown under each patient sequenced. C is reference and T is p.Thr267Met variant

The Institutional Review Board of UCLA approved all methods for this study. The family was identified during routine clinical evaluation in their native country of New Zealand. The affected sisters (Fig. 1) exhibited non-progressive ataxia with onset from early infancy. The oldest sister was observed to have early delated motor milestones and first walked with crutches at age 7 years. Speech was also delayed with first word at age 5.5 years. By school age, learning difficulties were noted and formal assessment of IQ was 54. Neurological examination as an adult in her mid-forties was notable for strabismus, horizontal nystagmus, and hypermetric saccades to the left with hypometric saccades to the right (saccades were mildly misdirected). Speech exhibited a scanning dysarthria. Motor and sensory systems were intact although tone was decreased and there was a mild tremor. Limb and gait ataxia were present. MRI of the brain showed atrophy of the cerebellum. The younger sister also had early delated motor milestones and first walked with crutches at age 3.5 years. Speech was also delayed with first word at age 3 years. By school age, mild learning difficulties were noted as well and IQ was measured at 73. Neurological examination as an adult in her early-forties was similar to that her sister. MRI of the brain was not performed.

## Diagnostic evaluation

The family received comprehensive clinical evaluations for acquired causes of ataxia [7] and, after genetic counseling, provided written informed consent for participation in this research study. The two affected sisters tested negative for common genetic ataxias (SCA1, SCA2, SCA3, SCA6, SCA7, and SCA36). Genomic investigation for causes of spinocerebellar ataxia was performed using whole exome sequencing (WES) on all four members of the family (Fig. 1). The Nextera Rapid Capture Exome Kit (Illumina, San Diego, CA) was used to prepare the genomic DNA (gDNA) libraries. gDNA libraries were sequenced on the HiSeq 2500 sequencer in the rapid-run mode (Illumina, San Diego, CA) as 107-bp paired-end reads. Burrows-Wheeler Aligner (BWA) [8] was used to align sequencing data to the hs37d5 reference and SAMtools [9] was used to post-process the alignment data. Picard Tools (https://broadinstitute.github.io/picard/) was used to compute sequence alignment statistics and marked duplicate reads. Variants were called based on the Broad Institute’s Genome Analysis Toolkit (GATK) version 3 best practices [10, 11]. Family relationships were confirmed by the relatedness algorithm from VCFtools [12]. Variants were annotated with VarSeq (Golden Helix, Inc., Bozeman, MT, www.goldenhelix.com). Variants were classified based off the American College of Medical Genetics and Genomics (ACMG) guidelines [13].

Exome sequencing identified a pathogenic variant in the *ITPR1* gene present in both affected sisters. The *ITPR1* variant (hg19:chr3:4687357C > T, p.Thr267Met) was previously reported as occurring de novo or sporadically [3–5] and is not present in the ExAC (exac.broadinstitute.org) or gnomAD (gnomad.broadinstitute.org) public databases of human variation. In HEK293 cells [5] and in IP_3_R triple knockout HeLa cells [6], the p.Thr267Met variant showed reduced IP3-induced Ca^2+^ release suggesting it is a loss of function mutation. Although observed in multiple families [3–5], this variant has not previously been reported as inherited through the germline. WES in our data indicated that the variant was present at low level (2/242 reads) in the unaffected mother suggesting she is germline mosaic for the variant.

## Discussion and conclusions

There is little published information about genomic variation in the New Zealand Māori population and the prevalence of spinocerebellar ataxia in this population has not been fully studied. To date, there have been 6 reported families with Māori ancestry and spinocerebellar ataxia [14–16]. Thus far, patients have been reported with cerebellar ataxia, neuropathy, and vestibular areflexia syndrome (CANVAS) [14], hereditary spastic paraplegia type 7 [15], and autosomal recessive spastic ataxia of Charlevoix-Saguenay [16]. Mutation of the *ITPR1* gene is associated with two distinct ataxic phenotypes, a progressive adult-onset form characterized primarily by gait and limb ataxia, termed Spinocerebellar Ataxia Type 15 (SCA15), and a congenital non-progressive form associated with intellectual disability (SCA29) [1, 4]. Here, we provide the first report of SCA29 in a family of Māori ancestry originating from New Zealand. The identified p.Thr267Met mutation was germline inherited from the unaffected Māori mother who is mosaic. The observation of this same variant occurring in multiple families of different ethnic origin, including now the Māori population of New Zealand, suggests this site may be a mutational hotspot within *ITPR1.* This is supported by a recent study that identified this variant as part of a rare cluster of missense mutations found within the *ITPR1* gene [17]. Our findings expand the prevalence and underlying genetic etiology of SCA29. Given the absence of ataxia in other members of the large extended maternal family, we speculate that this mutation occurred within the mother during her early development, resulting in the presence of the mutation in the germline and at a low level in the blood and perhaps other tissues, but sparing her of the neurological phenotype. We have previously observed parental mosaicism in other SCA29 families, both maternally (p.Gly2506Arg) [4] and paternally (p.Arg269Trp, unpublished observation), suggesting that it may be clinically informative to assess the parents of a child with SCA29 for mosaicism to aid in appropriate genetic and reproductive counseling.

## Data Availability

The datasets generated and/or analyzed during the current study are not publicly available as they could compromise the anonymity of the subject but specific data elements may be available from the corresponding author on reasonable request.

## Abbreviations

ACMG: American College of Medical Genetics and Genomics
BWA: Burrows-Wheeler Aligner
GATK: Genome Analysis Toolkit
gDNA: Genomic DNA
ITPR1: Inositol 1,4,5-Trisphosphate Receptor Type 1
SCA: Spinocerebellar Ataxia
SCA29: Spinocerebellar Ataxia Type 29
WES: Whole Exome Sequencing
